# Integration of antenatal syphilis screening in an urban HIV clinic: a feasibility study

**DOI:** 10.1186/s12879-014-0739-1

**Published:** 2015-01-13

**Authors:** Yukari C Manabe, Gertrude Namale, Elizabeth Nalintya, Joseph Sempa, Rosalind Parkes Ratanshi, Nadine Pakker, Elly Katabira

**Affiliations:** Infectious Diseases Institute, Makerere College of Health Sciences, PO Box 22418, Mulago Hospital Complex, Kampala, Uganda; Division of Infectious Diseases, Department of Medicine, Johns Hopkins University, Baltimore, MD USA; Amsterdam Institute for Global Health and Development, Amsterdam Medical Center, Amsterdam, Netherlands; Department of Medicine, Makerere College of Health Sciences, Kampala, Uganda

**Keywords:** Antenatal, Syphilis, Integration, HIV, Partner testing

## Abstract

**Background:**

Syphilis infection during pregnancy leads to avoidable morbidity and mortality and remains a significant problem in sub-Saharan Africa. Despite global initiatives to increase the proportion of pregnant women screened, implementation has been slow. We sought to investigate the feasibility of adding syphilis screening within an integrated antenatal HIV clinic.

**Methods:**

Pregnant women attending the HIV antenatal clinic were sequentially enrolled and consenting participants answered a questionnaire on sexual behavior and previous pregnancies, provided sociodemographic data, and were tested using rapid plasmin reagin (RPR). If positive, participants were treated with benzathine penicillin. All were given a partner notification slip and were followed up after delivery to determine birth outcomes.

**Results:**

584 of 606 (95.7%) women approached and consented to test for syphilis. 570 women were enrolled (median age 29 (IQR 25–32) with a median (IQR) CD4 of 372 (257–569) cells/μL). Of the 5.1% (29/570) with a positive RPR, all were asymptomatic, were successfully contacted, and treated with benzathine penicillin without adverse reactions. Overall, 61 (12.1%) of the participants had an adverse birth outcome. In the bivariate analysis, only age was significantly different between those with and without a positive RPR (RR = 1.15, 95% CI 1.065-1.248; p < 0.001). Partners of only 10 (34.5%) participants returned for treatment.

**Conclusions:**

Structural interventions such as opt-out testing for syphilis within integrated HIV-antenatal care clinics are feasible and capitalize on the excellent care programs that have already been established for HIV care. Novel approaches are required for partner notification.

## Background

Pregnant women who are infected with *Treponema pallidum,* the causative bacterial organism of syphilis, are at high risk for adverse birth outcomes. It is estimated that without screening and treatment, 53-82% of these women will have negative outcomes including late abortion, stillbirth, prematurity, low birth weight, neonatal death, and congenital syphilis [1]. In sub-Saharan Africa (SSA), there are approximately 535,000 pregnant women with active syphilis yearly [2]. A meta-analysis by Hawkes and colleagues estimated that the antenatal syphilis screening interventions could reduce the incidence of syphilis-related perinatal death and stillbirth by ~50% globally with the majority of the benefit in low and middle-income countries [3,4]. However, despite the launch of the World Health Organization (WHO) global initiative to eliminate congenital syphilis [5] and widespread policies for syphilis screening in most developing countries, implementation of syphilis screening has been slow due to inadequate and centralized laboratory services [6]. Global estimates from the WHO showed wide variation among African countries with 34% to >95% of women accessing antenatal care of which 3% to >95% of pregnant women were screened for syphilis [7].

HIV clinics offer opportunities to screen for other prevalent infectious diseases. As a regular point of contact between health care workers and patients, integration in these clinics can maximize the public health effect of limited resources. As sub-Saharan African countries implement prevention-of-mother-to-child transmission (PMTCT) programs such as option B (antiretroviral therapy (ART) to pregnant women at any CD4 count for pregnancy and breast feeding) and option B plus (ART to pregnant women for life at any CD4 count) in accordance with the 2013 WHO guidance [8], HIV clinics are becoming more frequently involved in providing integrated antenatal and HIV care. This integration of sexual and reproductive health (SRH) services in HIV care provides an opportunity to offer other services such as family planning, sexually transmitted infection and cervical cancer screening within the HIV clinic setting. However, there is very little operational research data on outcomes and processes of integrating SRH services into mainstream HIV care. The WHO recently endorsed integrated syphilis and HIV screening in antenatal clinics for the elimination of mother-to-child transmission of both syphilis and HIV [9].

In Uganda, it is estimated that 1.8% of pregnant women are infected with syphilis based on a country wide serosurvey, however less than 2% of pregnant women are tested in pregnancy [6,10]. Following success in establishing an integrated tuberculosis-HIV clinic which reduced tuberculosis treatment default [11], we sought to integrate SRH care into our HIV services. An early focus was to extend syphilis testing to all pregnant women attending an integrated antennal HIV clinic. This paper describes the feasibility of an integrated approach to syphilis screening within an antenatal HIV clinic and highlights the major barriers to wider roll-out and implementation.

## Methods

### Study setting and design

We performed a descriptive cohort study of consecutive, HIV-infected pregnant women who were followed at the Infectious Diseases Institute (IDI) in Kampala, Uganda. IDI is a clinical and research center of excellence that sees more than 350 patients per day, has approximately 9,000 patients enrolled in HIV care and more than 7,000 on ART. Patients were prospectively recruited and investigated upon referral to the antenatal clinic which is integrated within the adult IDI HIV clinic. Four weeks prior to delivery, women transferred care to the Mulago National Referral Hospital antenatal outpatient clinic. All participants provided written informed consent and the study was approved by the Makerere University Research and Ethics Committee as well as the Uganda National Council of Science and Technology. Patients were eligible for the study if they were confirmed to be pregnant, and were registered at the IDI clinic.

### Procedures

Demographic characteristics were recorded and a standardized questionnaire was completed, documenting sexually transmitted infection symptoms, sexual behavior, previous birth outcomes, relevant obstetric and prenatal histories, and most recent CD4 T cell count. Each patient provided a venous blood sample for non-treponemal assay testing (rapid plasma reagin, RPR, Taytec, Canada) in the clinic stat laboratory. RPR was the Ugandan standard at time of the study. All women who were confirmed to be RPR-positive were contacted to return to clinic and were treated with one intramuscular injection of 2.4 million units benzathine penicillin per the Ugandan national treatment guidelines for syphilis. Mothers who were penicillin-sensitive were treated with erythromycin 500 mg four times per day for ten days. All patients with a positive RPR were given an anonymized partner notification slip and encouraged by the nurse counselors to invite their partners to return to the IDI clinic for free treatment. Risk of reinfection was emphasized. The slip explained to the partner that they may have been exposed to a sexually transmitted infection and that they should seek medical follow-up.

Women with syphilis were followed up according to the Ministry of Health recommended routine antenatal schedule, although many patients were seen more frequently if they were on ART for HIV, for example. Participants were either contacted by phone within 30 days of the estimated due date to follow up on pregnancy outcome or reviewed by a clinician at the IDI clinic if they had a return appointment scheduled. Participants were not monetarily compensated for their time.

Treponemal antibody testing (Determine Syphilis TP, Alere, Japan) was then retrospectively performed as batched testing in the Makerere University-Johns Hopkins University clinical core laboratory which is College of American Pathologists (CAP) certified and undergoes external proficiency testing as well as on-going daily internal quality assurance procedures.

The data was double data entered by data entrants and analyzed by STATA (Stata Corp. STATA 11.1, College Station, Texas, USA). Descriptive statistics were used for the demographic and obstetric characteristics. Patients with a positive RPR test were compared to those who tested negative using chi-squared test and Wilcoxon rank sum test.

## Results

### Patient characteristics

**Figure 1:**
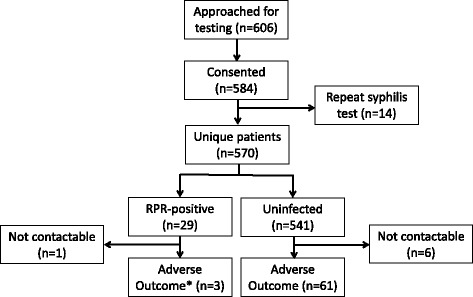
**Study enrollment diagram.**

Patients were recruited from October, 2010 through December 2011 (Figure 1). Six hundred and six consecutive, pregnant, HIV-infected women were approached to enroll in the study within the IDI antenatal clinic. Five hundred eighty-four (95.7%) women consented to participate and underwent syphilis testing; 14 were tested on 2 occasions. Among the 570 unique participants, the median age was 29 years (IQR 25–32), 93.5% (533) had either a primary or secondary school education, 65.8% (375) of women were ever married with 30.7% (175) currently living with their spouse. The majority of the women were employed (59.1% (337)). Most of the participants presented for syphilis testing in the first or second trimesters (418/570, 73.3%) and 15.0% (86) were primagravida (Table 1). Twenty-two participants presented to the HIV clinic for the first time at the time of pregnancy.

**Table 1:** **Demographic characteristics of the participants**

**Variable**	**Syphilis stage blood test**			**P-value**
	**RPR-negative (n = 541)**	**RPR-positive (n = 29)**	**Total (n = 570)**	
Age of respondents: median(IQR)	29(25–32)	25(22–27)	29(25–32)	<0.001
CD4 count at diagnosis: median(IQR)	400(274–577)	433(255–553)	402 (272–575)	0.867
On ART at time of syphilis testing	324 (59.9)	14 (48.3)	338	0.211
NNRTI-based	264(81.4)	11(78.6)	275(81.3)	
PI-based	55(17.0)	3(21.4)	58(17.1)	
NRTI-based	5(1.5)	0	5(1.5)	
Duration since start of ART (weeks)	101.1(5.1-209.6)	28.9(2-89	93.8(4.8-208.4)	0.265
BMI	23.1(4.2)	21.3(2.9)	23.0(4.2)	0.118
Number of previous pregnancies	460	24	484	0.341
1	152(33.0)	10(41.7)	162(33.5)	
2-3	216(47.0)	12(50)	228(47.1)	
≥4	92(20.0)	2(8.3)	94(19.4)	
Level of education				0.423
none	34(6.3)	3(10.3)	37(6.5)	
primary	222(41.0)	14(48.3)	236(41.4)	
secondary	285(52.7)	12(41.4)	297(52.1)	
Marital status				0.437
single	20(3.7)	0	20(3.5)	
ever married*	357(66.0)	18(62.1)	375(65.8)	
co-habiting	164(30.3)	11(37.9)	175(30.7)	
Employed	321(59.3)	16(55.2)	337(59.1)	0.657
Had sex in the last 1-month	408(75.4)	22(75.9)	430(75.4)	0.957
Partner agrees to use a condom	326(79.9)	16(72.7)	342(79.5)	0.417
Used a condom during the last sexual encounter	254(62.2)	13(59.1)	267(62.1)	0.773
Previously treated for STI's	67(12.4)	4(13.8)	71(12.5)	0.823
Received PMTCT	297(54.9)	12(41.4)	309(54.2)	0.182
Hospitalized during this pregnancy	6(1.1)	1(3.4)	7(1.2)	0.308
Hypertension during this pregnancy	2(0.4)	1(3.4)	3(0.5)	0.145

*includes married, separated/divorced, and widowed.

The median CD4 of the participants overall was 402 (IQR 272–575) cells/μL. For the more than half (54.2%) of participants on ART, the median (IQR) CD4 was 372 (257–569) cells/μL and 426 (299–598) cells/μL for participants not on ART. Of the women taking ART, 81.4% (275) were on a non-nucleoside reverse transcriptase inhibitor (NNRTI)-based regimen, 17.1% (58) on a protease inhibitor (PI)-based regimen, and 1.5% (5) on nucleoside reverse transcriptase inhibitor (NRTI)-based regimen only.

### Prevalence and factors associated with a positive RPR in pregnant HIV-infected women

A positive RPR was found in 5.1% (29/570) of the participants, none of whom were symptomatic for primary or secondary syphilis. All 29 were successfully contacted and treated with benzathine penicillin and did not have adverse reactions. Twenty-eight of the 29 participants with a positive RPR were able to be contacted post-partum. Three (10.7%) of the RPR-positive participants had spontaneous abortions, and no stillbirths, premature births, or early deaths. In the RPR-negative group, 505 (of 541, 93%) participants were able to be contacted post-partum. Of these, 17 (3.4%) had a premature birth, 23 (4.6%) had a spontaneous abortion, 11 (2.2%) had a still birth, and 10 (2.0%) participants had the baby die within 7 days of birth. Overall, 61 (12.1%) had an adverse birth outcome. In the bivariate analysis, only age was significantly different between those with and without a positive RPR (RR = 1.15, 95% CI 1.065-1.248; p < 0.001); RPR positive participants tended to be younger.

For the 84.9% (484/570) of the participants who had previously been pregnant, 5.0% (24/484) were RPR-positive this pregnancy. There were no adverse birth events for those with a positive RPR test. Only 2.8% of the patients with a negative RPR reported a previous adverse outcome. 62 (12.8%) had been treated previously for sexually transmitted infections. Confirmatory testing with a treponemal antibody was done retrospectively and found only 34% (10/29) were positive by the confirmatory test with an overall rate of confirmed syphilis of only 1.8% (10/570).

### Uptake of syphilis screening in partners of RPR-positive patients

Of the 29 participants with a positive RPR test, only 17 (58%) agreed at time of treatment to notify their partners. Of these, only 10 (58.8%) partners returned for counseling, testing and treatment.

## Discussion

Overall rates of syphilis in our study were comparable to the Uganda Bureau of Statistics AIDS indicator survey of 2011 [12] and antenatal syphilis prevalence rates reported in other Ugandan studies [10,13]. By integrating the syphilis screening into routine antenatal care in our HIV clinic, we achieved widespread screening of syphilis in pregnancy with no requirement for additional human resources. All women who were found to be positive were notified and received treatment, thereby potentially avoiding syphilis-associated maternal and infant morbidity and mortality. However, the number of women with a positive test was not large enough to show an impact on birth outcomes. Among the women with syphilis who had previously been pregnant, none had received syphilis treatment. However, none of those previous births had an adverse outcome by participant recall.

As well as increased syphilis testing, our experience of integrating sexual and reproductive health into HIV care also increased the proportion of women who were offered PMTCT option B amongst those not already on ART (data not shown). Likewise in Zambia, combined research and service delivery programs had a positive influence on RPR testing (OR 2.5 (95% CI 2.1-3.0)) [14], and in Mozambique, a longitudinal study showed that integrated efforts of syphilis screening into antenatal care was superior to a vertical program in increasing nationwide rates of syphilis screening among pregnant women [15].

In our resource-limited setting, a possible barrier to testing is the availability of validated methods of syphilis testing that are easy to perform and easy to interpret. There has been a long standing debate about non-treponemal test (RPR) as the screening test of choice for antenatal syphilis; RPR is time-intensive, requires lab expertise to perform, and electricity for a rotator. There is also a high false positive rate and, ideally, RPR positive patients should have a confirmatory treponemal test. However, more specific treponemal tests remain positive for life, which can overestimate active syphilis and may lead to overtreatment of pregnant women where resources are scarce. Conversely, treponemal tests cannot differentiate between an old treated infection and reinfection, which is also a challenge in sexually active pregnant women. The RPR false positivity rate was unusually high in our study suggesting biologic false positives due to pregnancy [16], HIV [17], or other mechanisms including uncontrolled temperature and humidity in the stat lab where the RPRs were performed. Unfortunately in our setting quantitative RPR titers are prohibitively expensive and, therefore, were not performed. In our cohort, we used traditional lab-based RPR testing, but did still manage to have a good uptake of syphilis testing and treatment implemented by HIV clinicians.

In our study, 75% of women had sex in the last month and only 38% of these did not use a condom. However, despite intensive counseling, we found that it was difficult to get participants with syphilis to notify their partners (58%). Ultimately only one-third of the partners came in for testing and treatment. Our intensive counseling did result in higher uptake than has previously been reported in the literature. In an implementation study in Uganda, <1% of partners of pregnant women who were offered testing ultimately came to clinic for HIV and syphilis testing [18]. In another evaluation in Zambia and Uganda in both rural and urban sites, 9.9% of men who were invited to test did so [10]. This has important implications for the roll-out of rapid syphilis testing and treatment, as reinfection (especially before delivery) is a serious concern in pregnant women. Use of treponemal tests in this situation is also debatable due to the inability to detect reinfection. In another assessment of routine syphilis screening in Mozambique, low rates of partner notification were also reported [15]. Integrated care led to significant screening in first and second trimester, although we acknowledge that we should also have introduced a retesting strategy for later trimesters. Intensive counseling combined with repeated notifications of the pregnant woman may increase partner testing rates. We are currently undertaking a study to assess novel strategies to address this issue (clinical trials.gov NCT02262390).

Authors from a recent review have suggested that the rapid elaboration of new point-of-care (POC) tests to encompass screening for multiple STIs may be both warranted and feasible [13]. The introduction of rapid, POC treponemal antibody testing will allow for screening in more peripheral facilities because the immunochromatographic tests strips detect serum antibodies to recombinant *T. pallidum* specific antigens and are reliable, rapid (8 minutes) and do not require refrigeration or specialized equipment [19-22]. Data from South Africa show that rapid testing may also increase the proportion of patients who test and are treated for syphilis [23]. The structured introduction of POC syphilis testing will make antenatal syphilis screening even more accessible as it will circumvent challenges in laboratory infrastructure needed for RPR testing in resource-limited settings [6,10,24]. It will make implementation of STI screening easier for health care workers in resource limited settings, and will enable same day results, which can assist in uptake of treatment. There are currently 5 manufacturers that have integrated HIV and syphilis testing in the same POCT test (23) and once validated and widely available, these will be an additional step in the integration of SRH activities. Challenges remain in the proportion of women who access antenatal care throughout sub-Saharan Africa [25], but with new PMTCT initiatives this should improve. We need to seize the opportunity of this roll out, and the training of health care workers during this period to integrate syphilis screening with HIV testing [26] into PMTCT B and B plus programs.

## Conclusion

Structural interventions such as opt-out testing for syphilis integrated within HIV services capitalize on the excellent vertical care programs that have already been established for HIV care and are feasible.

## Abbreviations

ART: Antiretroviral therapy
HIV: Human immunodeficiency virus
IDI: Infectious Diseases Institute
IQR: Interquartile range
NNRTI: Non-nucleoside reverse transcriptase inhibitor
NRTI: Nucleoside reverse transcriptase inhibitor
PI: Protease inhibitor
PMTCT: Prevention of mother-to-child transmission
POC: Point-of-care
RPR: Rapid plasmin reagin
RR: Relative risk
SRH: Sexual and reproductive health
SSA: Sub-Saharan Africa
WHO: World Health Organization
